# Principles and processes behind promoting awareness of rights for quality maternal care services: a synthesis of stakeholder experiences and implementation factors

**DOI:** 10.1186/s12884-017-1446-x

**Published:** 2017-08-31

**Authors:** Asha S. George, Casey Branchini

**Affiliations:** 1 South African Research Chair in Health Systems, Complexity and Social Change, School of Public Health, University of the Western Cape, Private Bag X17, Bellville, 7535 South Africa; 2 Health Systems Program, Department of International Health, Johns Hopkins School of Public Health, 615 North Wolfe Street, Baltimore, MD 21205 USA

**Keywords:** Rights, Health promotion, Maternal health, Implementation, Context, Stakeholder perspectives

## Abstract

**Background:**

Promoting awareness of rights is a value-based process that entails a different way of thinking and acting, which is at times misunderstood or deemed as aspirational.

**Methods:**

Guided by the SURE framework, we undertook a secondary analysis of 26 documents identified by an earlier systematic review on promoting awareness of rights to increase use of maternity care services. We thematically analysed stakeholder experiences and implementation factors across the diverse initiatives to derive common elements to guide future efforts.

**Results:**

Interventions that promote awareness of rights for maternal health varied in nature, methodological orientation, depth and quality. Materials included booklets, posters, pamphlets/ briefs and service standards/charters. Target populations included women, family members, communities, community structures, community-based and non governmental organizations, health providers and administrators, as well as elected representatives. While one initiative only focused on raising awareness, most were embedded within larger efforts to improve the accountability and responsiveness of service delivery through community monitoring and advocacy, with a few aiming to change policies and contest elections. Underlying these action oriented forms of promoting awareness of rights, was a critical consciousness and attitudinal change gained through iterative capacity-building for all stakeholders; materials and processes that supported group discussion and interaction; the formation or strengthening of community groups; situational analysis to ensure adaptation to local context; facilitation to ensure common ground and language across stakeholders; and strategic networking and alliance building across health system levels. While many positive experiences are discussed, few challenges or barriers to implementation are documented. The limited documentation and poor quality of information found indicate that while various examples of promoting awareness of rights for maternal health exists, research partnerships to systematically evaluate their processes, learning and effects are lacking.

**Conclusion:**

Rather than being aspirational, several examples of promoting awareness of women’s rights for quality maternity care services exist. More than mainly disseminate information, they aim to change stakeholder mindsets and relationships across health system levels. Due to their transformatory intent they require sustained investment, with strategic planning, concrete operationalization and political adeptness to manage dynamic stakeholder expectations and reactions overtime. More investment is also required in research partnerships that support such initiatives and better elucidate their context specific variations.

## Background

Women’s rights in pregnancy and childbirth were agreed to in 1994 at the International Conference on Population and Development [1]. Subsequently, these rights were endorsed by multiple United Nations agencies and bodies, donors and civil society [2–6]. Despite this affirmation, women still struggle to claim their sexual and reproductive rights, including those related to maternity care services. Notwithstanding overall declines in maternal mortality, skilled attendance at birth remains one of the most glaring inequalities in global health [7]. Even when women do access maternity care services, the disrespect and abuse they face in those services is an extreme violation of their rights [8].

In a previous systematic review, we assessed the effects of interventions that promote awareness of rights on the use of maternity care services in low and middle income countries (LMICs) [9]. We identified four studies promoting awareness of rights that reported health outcomes with clear research methods detailing increases in antenatal care [10–12] and facility births [11–13]. Improvements in human rights measures such as availability, acceptability, accessibility, quality of care, as well as the capacity of stakeholders were also reported to varying extents by these four studies.

Promoting awareness of rights is a value-based social process that often entails a different way of thinking and acting. Their efforts to transform health systems is at times misunderstood or seen as just aspirational. Shakespeare in Hamlet memorably stated ‘though this be madness, yet there is method in it’ [14]. We examine the diverse range of initiatives that promote awareness of rights to quality maternity care services to derive the common principles and processes related to stakeholder experiences and implementation factors to guide future efforts in this area.

## Methods

We undertook a secondary analysis of the documents identified through the systematic review [9]. Documents were identified through a systematic mapping of maternal health literature published from 2000 to 2012 that focused on health system and community-based interventions for improving maternal health and for reducing maternal health inequities in low and middle-income countries (LMIC) (http://eppi.ioe.ac.uk/webdatabases4/Intro.aspx?ID=11). In addition, we conducted additional searches for studies in Pubmed from 2010 to 2014, an internet search and contact with experts. Inclusion and exclusion criteria were developed to find studies across all populations with participants defined as pregnant women or women in labor and with interventions aimed to improve awareness of rights among women, men, community members or health workers and program administrators.

For this secondary analysis, apart from the four studies that reported health outcomes in the systematic review, we analysed an additional 18 documents identified through the systematic review as they described interventions promoting rights for maternal health and an additional four that promoted awareness of rights for other health areas [15–18]. Only the studies included in the systematic review [10–13] were assessed for quality using the Effective Public Health Practice Project quality assessment tool [19], designed to assess study quality of quantitative studies across various fields of public health (Table 1).

**Table 1:** Studies detailing interventions promoting awareness of women’s rights for maternity care services with reported specified health outcomes (*N* = 4)

#	Study	Study type and quality Document type	Setting	Nature of intervention/initiative/project	Reported health outcomes
1	Pandey, Sehfal, Roboud, Levine & Goyal 2007	-Cluster randomized trial -Strong quality Journal article	Uttar Pradesh, India Study in 105 village clusters across 21 districts; Intervention with 22,495 households in 55 village clusters across 11 districts	4–6 public meetings during two visits spaced 2 weeks apart to disseminate information about entitled health and education services and village governance	ANC increase
2	Bjorkman & Svensson 2009	-Cluster randomized trial -Moderate quality Journal article	Uganda Rural Uganda, 4 regions, 9 districts; Study included 50 communities. Intervention with approximately 55,000 households in 25 communities	A community score card process with a week of meetings when communities and health facility staff review local priorities and action plans and agree on contracts monitored by communities, revisited in meetings 6 months later	ANC increase (not statistically significant) Facility delivery increase
3	Ganju, Khanna, Taparia & Hardikar 2014	-Participatory action research -Weak quality Newsletter article	Gujarat, India Intervention with 10,374 people in 12 villages in two districts	Over 2 years local volunteers visit families and prospectively fill a monitoring tool for every woman once during pregnancy and once during post-partum. A report card is developed to dialogue with different stakeholders and support local action.	ANC increase Facility delivery increase
4	Sinha 2008	-Pre- and post-intervention design -Weak quality Working paper	Andhra Pradesh, India Intervention with approximately 40,000 people in 37 villages and poor area of headquarter village in 1 district;	Over 15 months awareness raising and community support for pregnant women through local government and youth committees; involvement of their families through monthly meetings; and home visits by a community organizer who worked with families to create a birth preparedness plan and support access to care.	ANC increase Facility delivery increase

Data was abstracted and discussed by two persons (AG and CB). Variables included the experiences of key stakeholders, their attitudes and responses to the intervention and contextual factors related to implementation. We conducted a narrative synthesis guided initially by the Supporting the Use of Research Evidence framework (SURE) developed by WHO to highlight relevant implementation and contextual factors [20]. Abstracted findings were reviewed and synthesized by the lead author (AG) following a process of constant comparison. After drafting synthesized findings, authors revisited original articles to check their interpretations.

## Results

### Study characteristics

The 26 documents that reported interventions that promote awareness of rights for maternal health and other related health areas (Tables 1 and 2) varied widely in nature, methodological orientation, depth and quality. Only four studies included health outcomes, with study quality ranging from strong to weak. Nine were peer reviewed journal articles that were either qualitative [16, 21–23]; used multiple data sources [24–26], or experimental studies [10, 13], with the remaining 17 documents being grey literature.

**Table 2:** Documents detailing interventions promoting awareness of women’s rights for maternity care services or rights for other health areas without reported specified health outcomes (*N* = 22)

#	Document	Document type and study type if applicable	Setting	Nature of intervention/ initiative/ project
1	Bajpai 2009	Report	Rajasthan, India 15 villages across 5 districts of Rajasthan, India	The project supported a Maternal Health Right Awareness program. They supported capacity building of community based organizations, women’s self- help groups, health committees, observed outreach maternal and child health days, improved linkages with frontline health workers and held public hearings. Booklets on maternal health entitlements were distributed, meetings held and street plays, folk theatre performed.
2	Burket, Hainsworth & Boyce 2008	Report	Angola, Ethiopia, Ghana, Kenya, Nigeria, Mozambique, Tanzania, Uganda	Pathfinder implemented youth friendly post-abortion care across the eight countries tailored to their specific contexts. Across all eight countries the project worked to improve facility level care through facility assessments, action plans, provider training, orientation meetings for the remaining staff, facility renovations and supportive supervision. In addition, community level work supported sensitization meetings and peer educator training. In particular, in Mozambique, youth associations undertook community mobilization and outreach to raise awareness on rights related to accessing post-abortion care.
3	CARE International & ICRW 2010	Report	Uttar Pradesh, India 2 districts in Uttar Pradesh, India	ISOFI (Inner Spaces Outer Faces Initiative) was from 2007 to 2010. Integrated into an existing maternal health program, interventions facilitated dialogues that explored personal values and challenged assumptions related to gender and sexuality. For example, pictorial flash cards that prompted discussion on men’s role as supporters for women’s rights, the division of household labor, domestic violence, and women’s rights to seek care with a skilled provider. The project also brought couples together in a public location to allow them to learn and discuss MNH care in a safe space and carried out media campaigns (e.g., puppet shows, theater, etc.) focused on gender-related discrimination. CARE also worked closely with district health staff to build community-level capacity. Examples include training for CHWs on counseling men on their role in MNH and the integration of iterative and open-ended exercises for discussion on gender and sexuality with district and sub-district health meetings.
4	CARE International 2012	Report	Bangladesh, Bolivia, DRC, India, Peru, Tanzania	The following case studies were documented: Bangladesh-Community Support Systems in two sub-districts from 2006 to 2010 that tracked every pregnant woman, supported community awareness and resources for maternal health, strengthened local governance mechanisms (based on the Dinajpur Safe Mother Initiative); India-Inners Spaces Outer Faces (ISOFI) see above; Bolivia- Participatory Community Management implemented for 1 year in four rural and peri-urban departments that tracked pregnant women and other types of service utilization, raised community awareness, supported community monitoring of services (based on FEMME program in Peru); DRC -Uzazi Bora Project in Kasongo district from 2007 to 2011 supporting local governance mechanisms, raising community awareness; Peru-FEMME program in Ayacucho region from 2000 to 2005, subsequent policy advocacy to scale up through FEMME+ from 2006 onwards; Tanzania - HEqP Initiative – highlighting inequities and rights violations; community scorecard process, community awareness, advocacy and policy work from 2007 to 2011.
5	Crump 2003	Report	Nepal, Mexico	Nepal newborn case study decided against following a rights approach because it brought in too much complexity, though it included Minister of Human Rights into their work. Findings conflict with other case studies on a rights-based approach to maternal health in Nepal; Mexico Safe Motherhood committee worked on a charter for patient’s rights and promoted reproductive rights in reviewing policies, working with other stakeholders, but mostly an overview case study; Indonesia Involvement of Minister of Human Rights in maternal health advocacy
6	Das & Dasgupta 2013	Report	Uttar Pradesh, India	Reflects on the experience of the Mahila Swasthya Adhikar Manch (MSAM) or Women’s Health Rights Forum in Uttar Pradesh, India, which raised women’s awareness of entitlements and supported their role in monitoring service delivery and dialoguing with policy makers to improve access to health services to improve maternal health.
7	Dasgupta 2011	Journal article Descriptive study	Uttar Pradesh, India	Describes the experiences of a NGO, SAHAYOG, developing ‘rights based’ strategies around maternal health. Uses recent frameworks on accountability and gendered rights to draw out lessons. Multiple initiatives discussed that raised awareness of rights: forums, campaigns, etc.
8	Davis-Floyd, Pascali-Bonaro, Davies & Gomez Ponce de Leon 2010	Newsletter	Includes examples from Bangladesh, Cambodia, Canada, China, Honduras, Ghana, India, Nepal, Romania, Uruguay.	Outlines 10 steps through which IMBCI aims to improve care throughout the childbearing continuum to save lives as well as prevent illness and harm from the overuse of obstetric technologies and promote health for mothers and babies around the world. Cites the approach as a “testament to and an affirmation of women’s fundamental rights during childbirth”. Cites the launching of a demonstration project in Canada and Uruguay.
9	DFID 2005	Guideline	Includes examples from Bangladesh, Cambodia, China, Ghana, Honduras, India, Nepal, Romania	Provides guidance to program managers specifically on how to put a human rights-based approach into practice and mentions specific case study examples.
10	DFPA 2010	Report	Bangladesh, India, Pakistan, Nepal	Supports civil society engagement for accountability in health governance Women’s Health and Rights Advocacy Partnership (WHRAP) with a focus on sexual and reproductive health and rights in India (Sahayog/Chetna), Pakistan (Shirkat Gah), Nepal (Beyond Beijing Committee), Bangladesh (Naripokkho) through ARROW based in Malaysia from 2003 to 2010. Targets vulnerable women in the most remote areas and applies 1) a rights-based approach recognizing that the rights are at all times relational between citizens (rights-holders) and the state (duty-bearers) and between international obligations and local citizens’ claims, 2) a policy engagement approach to increase the influence of civil society in political decision making in health. Community mobilization strategy involving community members and organizations in evidence production and in monitoring government accountability; an advocacy strategy to mobilize political will; an alliance strategy to build civil society coalitions; a boomerang strategy to leverage external and national actors.
11	Kayongo, Esquiche, Luna, Frias, Vega-Centeno & Bailey 2006	Journal article Descriptive study	Peru	AMDD and CARE began Femme project in 2000 focusing on 5 facilities improving EmOC and promoting a human rights approach in health care (specific effort to provide non-discriminatory care to local cultures, signs with information on health services, birthing chairs according to women’s preference, improved privacy during childbirth, microwave oven for hot food, name tags to address women by name, community human rights initiatives to demand accountability).
12	Kenney, Siupsinskas, Sharman, Adilbekova & Zues 2005.	Report	Kazakhstan	*ZdravPlus* is a health reform project, supported by USAID, which assists five Central Asian countries in providing effective and efficient health services through technical assistance to improve quality of care, strengthen the financing systems and management of health services, and enhance the population’s involvement in health care decisions. Interventions are linked to the context of de-medicalizing and rationalizing care, reducing the number of ANC visits, unnecessary examinations, tests, episiotomies, increasing attendance of partners at birth and empowering women to choose the position they give birth in, more individual vs. shared rooms, use of partograph, changing sterile to clean enough environment so that family members can more readily access laboring woman, increasing skin to skin, breast-feeding on demand, reducing extent of hospitalization.
13	Molina, Michelini, Escobar & Robinson 2010	Report Internal self-evaluation External qualitative study	Argentina	Evaluation of the ‘Child Rights Education for Professionals’ initiative, including in its annexes references to ‘Te Escucho’ a project promoting the rights of children and women within health.
14	Natoli, Renzaho & Rinaudo 2008	Journal article Qualitative study	Ethiopia	Lessons learned on reducing harmful traditional practices from the Adjibar Safe Motherhood project.
15	Papp, Gogoi & Campbell 2013.	Journal article Qualitative study	Orissa, India	Case study of efforts to improve accountability focusing on the role of local women, intermediary groups, health providers and politicians. It highlights three drivers of success: [1] generation of demand for rights and better services, [2] leverage of intermediaries to legitimize the demands of poor and marginalized women and [3] the sensitization of leaders and health providers to women’s needs.
16	Reis, Deller, Carr & Smith 2012	Report	19 countries	Outlines findings of RMC survey with key stakeholders about their experience implementing interventions to promote respectful maternity care (48 individuals, 19 countries). Discusses how safe motherhood initiatives must beyond the prevention of morbidity or mortality encompass respect for women’s basic human rights. Case studies outlining strategies to ensure that women are better informed of their SRHRs and how to exercise them.
17	Schooley, Mundt, Wagner, Fullerton & O’Donnell 2009	Journal article Qualitative study	Guatemala	Qualitative study of women’s support groups seeking care at Casa Materna; a maternity waiting home that provides prenatal, postnatal, infant and well women care inclusive of family planning
18	Shepard 2002	Book chapter Qualitative study	Peru	Qualitative case study documenting the experience of *Consorcio Mujer*, an initiative by several feminist NGOs, to work with communities and health providers in six municipalities. The first phase involved sharing the results of an evaluation showcasing violations of women’s rights when accessing health centers and initial dialogues about the results. The second phase supported trainings and meetings with both women in communities and providers separately, before bringing them together for dialogues, that would agree on action plans to improve quality of care in facilities in ways that would respect women’s rights.
19	SORAK Development Agency 2013	Report	Uganda	Community-based approach that empowers women with relevant knowledge and skills to demand and access care and commodities to exercise their rights. Discussion of SORAK’s projects (2011–2012) and key achievements. Of specific interest is the Women’s Maternal Rights Promotion Project, which is only touched upon briefly. The only indicator provided is process-level (number of women trained). Empowers women (particularly members of marginalized groups) to understand and claim their rights through the establishment of a complaint mechanism
20	Stoffregen, Andion, Dasgupta, Frisancho & Mutunga 2010	Report	India, Kenya, Peru	Field projects undertaken 2008–2009 to increase understanding of rights based approaches to maternal mortality reduction efforts by NGOs in three countries: SAHAYOG in India, FCI in Kenya and CARE in Peru. SAHAYOG supported the Mahila Swasthya Adhikar Manch (MSAM) or Women’s Health Rights Forum in two districts to document case studies representing women’s experiences of facility delivery and to discuss this with national policy makers as part of a ‘Voices from the Ground’ meeting; briefing kits for elected officials developed and distributed; public hearings with local officials and women facilitated; booklet that supported discussion meetings following Friere methodology with local NGOs and women members. FCI in coordination with government partners implemented the Right to Care project that conducted workshops with community and religious leaders and health providers on maternal health and rights that resulted in action plans to ensure women’s rights to maternal health. CARE worked through its DFID funded Participatory Voices Project in Azangaro and Ayaviri provinces of Puno to support capacity building workshops, alliance building among local civil society networks, community monitoring of services, dialogues with community leaders and local authorities responsible for health services.
21	Strecker, Stuttaford & London 2012	Journal article	South Africa	Evaluation of pamphlets developed on the right to health as a part of a broader action research effort supported by a Learning Network for Health and Human Rights between local universities and civil society organisations.
22	Srofenyoh, Ivester T, Engmann, Olufolabi, Bookman & Owen 2012	Journal article Descriptive study	Ghana	Quality improvement in a hospital where “Customer service was addressed including a patient’s right to respect, privacy, emotional support, pain relief, communication, and timely access to care. These elements were promoted through lectures, informal discussion, and bedside example. Satisfaction surveys are conducted to monitor progress. Staff members who demonstrate excellent customer care are recognized”.

Across the 26 documents, interventions took place in a broad range of geographical contexts spanning Central Europe, Asia, sub-Saharan Africa and Latin America, with some also in high-income countries [27, 28]. Two initiatives, those supported by SAHAYOG [24, 29–31] and ISOFI India [32, 33] were detailed in more than one document.

### Synthesis of study content

#### Diversity of interventions

Diverse mediums were used to raise awareness of rights through booklets, posters, pamphlets/briefs [10, 12, 16] and in some instances service standards, charters or monitoring tools [11, 13]. In addition, initiatives also raised awareness of rights through mass rallies, campaigns and broader communication efforts [12, 22, 28, 29].

While one intervention focused solely on disseminating information [10], most were embedded within larger efforts to improve the accountability and responsiveness of services through community monitoring and advocacy [11–13, 21, 22, 28, 30, 34]. One also supported women from marginalized communities to run for political office [29]. In addition to disseminating information, these broader efforts fostered critical consciousness and attitudinal change across all stakeholders through capacity-building and training; the formation or reformation of community groups; and facilitated dialogue and participatory exercises [3, 12, 21].

While many initiatives focused on women, they were not always the sole target group. Initiatives also engaged with men and other decision makers at the household level, community structures such as health committees, health providers and managers, as well as elected representatives [11, 12, 29].

In the following sections, we seek to better understand these initiatives raising awareness of rights for maternity care by examining the experiences of each key stakeholder, before analyzing cross-cutting implementation factors.

#### Women’s perspectives and experiences

Few documents assessed women’s perspectives about the interventions directly, but several did detail women’s sense of personal empowerment and increased self-esteem [12, 16, 21–23, 29, 30]. This change was reported not just for women seeking care, but also for female health volunteers from marginalised communities [33]. Valuing themselves and changing their views about their social background entailed revisiting deep- seated social norms, particularly for women from marginalized communities that were marked by gender and other social inequalities [11, 21, 22, 35]. Some women in India were also hard to reach and engage in group processes because they migrated to their natal homes for pregnancy and childbirth [11, 35].

Key implementation factors included supportive friends and family members for sustaining peer-based learning [3, 12, 22, 23, 33]. Support groups were also valued by women [12, 22, 23] as they provided a space for social bonding, changing social norms and building self-esteem. Having safe environments to practice rights through role playing and/or with family members to learn negotiation and two-way communication skills were valued. Women had to strategize to find the right time and way to approach key individuals or groups; sometimes using humor to address issues [22, 23]. Significant time, capacity-building and iterative efforts were needed to overcome barriers such as paternalistic norms and internalized gender bias [20, 22, 23, 29].

Beyond supporting women’s awareness and agency in affirming and negotiating health seeking behaviour with family members and providers at an individual level, the promotion of rights awareness also resulted in women’s leadership and collectivisation more broadly [29–31]. For example, in Peru, local women replicated trainings on the right to maternal health with their own community as a means of building women’s leadership capacity [22]. In Uttar Pradesh, India, women recognized that they needed to organize at various administrative levels if they wanted their voices to be heard [29].

#### Community perspectives and experiences

Moving beyond the capacity of individuals, communities as a collective entity also provided critical support in promoting awareness of rights and ensuring their translation into action. In Uttar Pradesh, India [29], Peru and Tanzania [32], women’s mobilization strategically linked with other community groups to gain further support and foster a broader collective consciousness supportive of women’s rights to quality maternity care. Despite these examples of community engagement and mobilization, very few documents examined community perspectives directly. Those that did so reported positive views [12].

Supportive implementation processes included community dialogues combined with participatory social analysis and critical reflection that helped communities clarify the root causes underlying maternal mortality and discuss issues that had previously remained unspoken or unaddressed [28–30, 32]. In Bolivia, health center tours by community representatives also oriented them to the context of health service provision [32]. Before embarking on such community processes, gaining buy-in from community leaders and other influential community representatives was emphasized [3, 12, 17, 30, 32].

Successful projects also drew on community organizers with prior community work experience and who were trusted by community members [11, 12, 18, 22, 23]. Ensuring a gender balance among organizers, volunteers or peer educators was highlighted as critical to reach both female and male members [17, 18]. Strong relationships between volunteers or community organisers and health services, characterised by familiarity, trust and frequent collaboration, helped to ensure that awareness of rights is linked to increases in utilizing services [12, 17, 32].

Several projects supported local health committees and other structures that provide oversight of health centers [3, 10, 12, 13, 28, 30, 32, 35], although functionality was a concern in some cases [10, 32]. In Uganda, community awareness of health committee roles and responsibilities, alongside reconstitution of health committees were linked to increased utilization of maternity care services [13].

Several documents emphasized breadth of participation across social groups [10, 13, 28, 32, 35]. Conversely, disseminating information to key groups, without further community dialogue, facilitation or mobilisation, failed to increase maternity care seeking among low caste community members in Uttar Pradesh, India [10]. Barriers to equitable participation ranged from internalised community biases to pressing livelihood needs [30]. Some of these barriers were overcome by holding separate meetings [13], facilitating individual and group discussions about male resistance [12, 23] or identifying champions from specific groups and providing them with capacity-building [32].

Divisions among communities due to political vested interests, whether by insurgents or by entrenched political parties, also challenged efforts to build critical consciousness with respect to rights [22, 29]. Community members in Uttar Pradesh, India, felt that their elected village leadership were unapproachable and monopolized development work [10].

#### Health Provider’s perspectives and experiences

In general, frontline providers over time saw initiatives that promote awareness of rights as positive, particularly when aligned with generating demand for appropriate services [12, 24, 30]. However, some health providers found it hard to accept challenges to their authority, viewpoints and routines [16, 30, 32]. Others interpreted rights narrowly, without understanding the root causes underpinning the challenges women face in seeking care or conversely felt that social change was a longer term process beyond their influence [21, 22].

Health worker resistance or lack of cooperation was also linked to their difficult work environments, which can be contrary to respecting women’s rights [3, 28, 30, 32]. Staffing shortages, limited supplies and inadequate equipment combined with large patient loads inhibited provider ability and motivation to support promotion of rights awareness [22, 28]. In India and Bolivia, advocacy by community representatives to improve local budget allocations and resolve supply chain problems, demonstrated to providers’ a mutual commitment to improving health [12, 32].

Some providers, while acknowledging service delivery challenges that are beyond their control, also recognized the need for a change in mindsets on their behalf [18, 21, 22, 28, 30, 32, 33]. Supporting attitudinal changes among providers was aided by reflecting on their own experiences as users [22]. Ongoing refresher trainings for health providers to build solidarity/ motivation, especially when interventions are against the mainstream and challenge discriminatory ideologies, was important [32, 33]. Initiatives also focussed on strengthening interpersonal skills, identifying a group of champions to sustain energy for change and using a team approach so that individual providers are not isolated [15, 17].

In Uganda, provider awareness of patient’s rights and of their performance being reviewed in local committee meetings was linked to increased responsiveness and utilization of services [13]. However, measures to work with providers to support women’s rights to quality maternity services were short lived if they were not combined with higher-level support and structural changes creating a broader enabling environment [15, 22, 24, 28, 32]. Local providers rarely had the authority to address vacancies or delays in supply chains. In addition, managerial policies in certain contexts pushed service delivery targets that were counterproductive to supporting women’s rights to quality maternity care [22].

#### Health administrators and policy makers perspectives and experiences

Only two documents reported views of government officials, which were positive in nature [21, 30]. In particular, in Orissa, India, government officials reported that public hearings were useful in fostering collective critical consciousness and action. They felt that women from marginalized communities as individuals on their own did not have the confidence to articulate service delivery grievances [21].

Even though health administrator and policy makers viewpoints are not well documented, the importance of involving government counterparts, particularly higher level health administrators, in all steps of the project was stressed [25, 28, 30, 33]. Without efforts to ensure policy maker understanding, decision makers in Peru were found to prioritize purely technical interventions over the capacity building and facilitation required to support awareness of rights [32]. Several projects also found buy-in from senior management to be variable [28] [30, 33, 35], even if health administrators and policy makers were appreciative of women and community members monitoring and highlighting service delivery problems [24]. To overcome such challenges, several initiatives stressed the importance of maintaining relationships with administrators and policy makers at all levels of the health system [11, 12, 28, 30, 33, 34] and across political party affiliation given changes due to turnover and elections [32].

Many initiatives stressed the importance of higher level policies that support community inputs in local health planning and service oversight, whether in terms of broad decentralization measures or specific quality improvement mandates [3, 22, 30]. The lack of policies, guidelines or protocols listing out the obligations of health administrators and providers in supporting awareness and responsiveness to women’s rights to quality maternity care was also cited as a challenge [28, 32].

#### NGO perspectives and contexts

All the initiatives documented were facilitated by NGOs and many reported factors that NGOs perceived as supportive or challenging to their efforts to promote awareness of rights. Apart from a few examples, the majority of the articles did not discuss how NGOs themselves benefited or were at risk from this work, despite being central actors facilitating such initiatives.

In the few documents that discussed NGO experience, the capacity of NGOs to support rights initiatives was reported to have improved overtime [30, 33]. NGO efforts to raise awareness of rights and concomitant advocacy also lead to increased visibility and credibility from state actors, media and community members [12, 24]. At the same time, NGOs also ran various risks. In India [24] and in Peru [22], feminist organizations found themselves at risk of being co-opted by government, when included in government initiatives over which they had no control and that even at times violated rather than supported rights.

When working on government initiatives to advance rights, NGOs in India, Columbia, Peru and Chile noted that government contracts were underfunded. As a result, unpaid staff time and other administrative resources were often not accounted for, resulting in overworked staff and organizational strain [22, 35]. In India, additional staffing and funding was integrated with existing personnel and program systems so that gender and rights methodologies were not seen as extra work with no corresponding support. Flexibility in funding was also essential in supporting local adaptations [35].

Managing strategic alliances across civil society networks and social movements were critical when negotiating with government counterparts [30, 32, 35]. Doing so was reported to be challenging in Peru, due to the politics of representation. Some NGOs tread a fine line when advocating for the interests of marginalized populations, without coming from such marginalized groups themselves [22].

#### Cross-cutting implementation considerations

This section reviews implementation factors that were not specific to particular stakeholders.

#### Characteristics of tools

Posting information on free services and using suggestion boxes [13] was found to be effective, while posters explaining patient rights were not effective on their own in Uganda [13] or seen as empty gestures in other contexts [3]. The visual attractiveness and relevance of print materials was an important part of their use in South Africa and in India [11, 16, 32]. Community monitoring is facilitated if checklists are kept simple with indicators that reflect community priorities and that are observable by them [13, 22]. In India, group exercises facilitating dialogue on gender and rights emphasized fun at appropriate instances [33]. NGO involvement in developing the materials and in subsequent training influenced their use of pamphlets, with those less involved being less likely to use the materials [16]. Most critical was the ability of materials and exercises to act as context specific tools to foster interactive learning and dialogue [16].

#### Strategic orientation

A process of vernacularisation was cited as being critical for local stakeholders to adopt rights principles [3, 16]. This ensures that rights are articulated in a manner that reflects the local context, enabling their internalisation and ownership by local stakeholders. Authors also stressed that this must not contravene the rights principles they are meant to promote.

Several projects noted the importance of engaging both communities and providers simultaneously to promote awareness of rights [11–13, 30]. Furthermore, some initiatives emphasized a phased approach. Ensuring that services can handle increased demand and offer quality services, where women are treated with dignity and are affirmed/ valued, before promoting rights awareness that directs women to access such services [17, 23]. In addition, efforts that pursued multi-level, stakeholder and sectoral pathways were likely to build synergies that sustain the promotion of rights over the long term [12, 18, 32, 33].

Negotiating strategic dialogues and alliances was not an easy or predictable process. It required preparing groups ahead of time, fostering a common language and clarifying rules to counter power imbalances [11–13, 22, 30, 32]. It was also not a straightforward process. As setbacks and backlash entailed iterative capacity building and restrategizing to emphasize common ground and mutual aims. Expectations needed to be constantly re-aligned for both community members and providers [13, 28, 32]. Frustrations at times undermined women’s and community members’ confidence in their ability to support change [16, 22].

#### Strategic planning and sustained concrete operationalization

To overcome such setbacks and to realize the deep social transformation that promoting rights awareness entails, several factors related to strategic planning and operationalization were noted, particularly for initiatives to progress from rhetoric to reality.

At the *community level*, many documents stressed the importance of understanding women’s realities and what matters most to communities as a starting point [3, 12, 13, 21, 22, 28, 30, 32]. Involving family and community members in the design of the intervention is one way to ensure that initiatives understand their perspectives. In addition, a careful assessment of the local context and advance preparation between various stakeholders is required prior to events such as public hearings are held, to ensure that grievances aired will be addressed and most importantly that there will be no reprisals [21]. At the level of *health services,* a *s*ituation analysis to understand the context in which providers work was essential [3, 17]. Several studies also recommended stakeholder analysis or political mapping to ensure that initiatives secure buy-in from key officials and critical stakeholders [15, 22, 30].

Project experiences also emphasized that rights awareness initiatives be grounded in concrete actions and operational plans, with adequate follow up, monitoring and evaluation to ensure that they do realize their goals and not be only aspirational [22, 28, 30, 32, 35]. Costing studies were found to be particularly helpful in supporting and scaling up initiatives in Tanzania and Peru [32]. In addition, continuity of funding and leadership over several years to achieve change at multiple levels is required [18, 24, 32] to sustain initiatives and continually refresh relationships, as key stakeholders, particularly those within government may change.

Without significant capacity and consensus building among stakeholders, these initiatives can fail and frustrate the participants involved. Time and capacity building through iterative processes that support changed attitudes and norms across all stakeholders was noted as being vital across several contexts [16, 21, 22, 29, 30, 32, 33, 35]. Other elements of success included strong leadership, strategic thinking, political adeptness and management skills to support interventions that entail a mind shift that often runs against prevailing social norms [15].

## Discussion

### Summary of evidence

Across the various initiatives documented, women reported a sense of empowerment and increased self-esteem. This was supported through group processes that broke down isolation, nurtured social bonding and critical reflection. Women’s leadership and collective organizing around health rights were also key. Challenges included low literacy and in India, the mobility of pregnant women to natal homes during the period of pregnancy and childbirth. Significant time and capacity building are required to overcome deep seated gender and other forms of internalized discrimination [36].

Communities play a critical role in supporting women’s rights to maternity care services [37, 38]. Community dialogues that supported critical reflection about the root causes undermining women’s rights to health were instrumental. Gaining buy-in from community leaders and selecting community volunteers that are trusted, gender balanced, familiar with the local context and have strong relationships linking communities to health care services are important. Working through existing community health and local governance structures, such as health committees was critical if they are functional [39]. In some instances these structures needed to be reconstituted. Throughout efforts need to ensure that community processes reflect marginalized women’s perspectives and not further marginalize them [36].

Health providers perceived initiatives to raise awareness of women’s rights to quality maternity care services as having instrumental value in increasing utilization of services, but also as an opportunity to receive feedback and improve service provision. Similar to what Filby et al. [40] found, resistance was due to a narrow interpretation of rights or due to poor working conditions that constrained responsive care. Efforts to change provider attitudes included reflecting on their own experiences as patients, trainings focussed on interpersonal skills, identification of champions to model positive behaviour and a team approach to ensure against demoralisation. To be effective such initiatives need support from higher level managers and administrators, who reinforce standards and allocate resources necessary for responsive care.

While close collaboration with government counterparts was emphasized to mainstream interventions within the health sector, understanding and buy-in from senior level administrators and policy makers was not always found. Linkages to decision makers at all levels of the health system and across political party lines was important to ensure continuity of efforts, particularly when considering turnover of administrators and instability of political appointees. Ensuring policy recognition and mandate was also critical in enabling community dialogue and inputs into local service delivery for women’s rights, just as it is for community participation more broadly [39].

All initiatives documented were facilitated by NGOs, which in many instances led to increased capacity and visibility for them. At the same time, many NGOs also ran risks as they were under resourced, with insecure or inflexible funding, resulting in teams that were strained or not as well prepared as needed. When integrating such efforts to existing health projects, additional funding and staff may be required. As mentioned by Marston et al. [38], the legitimacy of NGOs also needs to be carefully balanced with representation of marginalized groups they serve. Strategic alliances across civil society and social movements were critical in ensuring broad based support for efforts to raise awareness of and respond to women’s rights to quality maternity care services.

Tools and materials used to raise awareness of rights need to be accessible, attractive and relevant to community members and must also foster opportunities for interactive learning and dialogue. Efforts to raise awareness of rights need to be grounded in the contexts of participants and internalised by them, without at the same time contravening their universal core principles. Initiatives in the health sector need to engage at various levels reflecting the multi-dimensional and multi-stakeholder character of women’s rights to quality maternity care services. It entails supporting dialogue and balancing power across stakeholders, whether at the individual level among women, at the interpersonal level with families and communities, at the service delivery level with providers and administrators and at the policy level with higher level decision makers. It requires time, capacity building and iterative facilitation to overcome resistance, backlash and setbacks [36]. Careful implementation with sound situational analysis, planning, budgeting, strong leadership, strategic thinking, political adeptness and good management are required.

Table 3 lists the stakeholder experience and implementation factors documented by the four studies that detailed interventions promoting awareness of rights and reported increases in antenatal care and facility births. These experiences and factors are only a subset of those elements reported in the larger set of documents examined. While several factors at community level are detailed, information on women, health provider and NGO experiences are notably scarce across these four studies. Few negative experiences or challenges are discussed. Furthermore, in this subset of studies study quality was inversely related to information related to stakeholder experience and implementation factors. This is partly because the high quality studies were published in journals that emphasize succinct reporting and do not emphasize elements related to implementation or stakeholder experiences.

**Table 3:** Stakeholder experience and implementation factors in studies detailing interventions promoting awareness of women’s rights for maternity care services with reported specified health outcomes (*N* = 4)

Supportive implementation factors	Challenging implementation factors	Studies			
		Pandey et al. 2007	Björkman & Svensson 2007	Ganju et al. 2014	Sinha 2008
Stakeholder Experience And Implementation Factors					
Women					
Increased awareness and self-esteem					X
Supportive friends and family members, peer-based learning					X
Support groups that provide a space for social bonding, discussion, breaking down isolation, building social ties, changing social norms					X
	Low literacy among women, migration of pregnant women			X	
Community					
Support from community leaders					X
Volunteers with prior experience working in intervention communities and trusted by community				X	X
Volunteers with strong relationships with health services enabling better linkages between communities and health services					X
Community awareness and support; participatory analysis and community dialogue combined with critical reflection and social analysis to identify hidden issues and underlying, root causes				X	X
Peer pressure to prevent harmful practices by traditional healers, violence against women, early marriage, dowry					X
Strengthening existing community governance structures related to health and if they are not functional, either dissolving them or creating parallel mechanisms to ensure community voice			X	X	X
Increased frequency of health committee meetings, although community members were not aware of this		X			
Community awareness of health committee roles and responsibilities			X		
Community action to improve inputs for local health care services fostering a sense of mutual commitments to improving health				X	X
Explicit equity considerations: Separate meetings within communities to ensure representation of interests by marginalized groups; Tailored capacity-building and accompaniment processes; Identification of champions from among the most poor and marginalized.	Social inequality, caste hierarchies, gender discrimination	X	X		
	Feeling by community members that sharing information on entitlements was futile, incomprehensible or fearful	X			
	Vested interests from local elected representatives that are unresponsive to community development needs.	X			
Health Providers					
Health provider knowledge about patients’ rights			X		
Health providers awareness that their performance was being discussed at local council meetings			X		X
Health Administrators And Policy Makers					
Relationships between individuals across levels of the health system				X	X
Non Governmental Organisation					
Additional capacity building, credibility and visibility for non-governmental organisation					X
Cross-Cutting Implementation Considerations					
Characteristics of tools					
Posting information on free services and use of suggestion boxes were effective, in contrast to posters on patient’s rights and obligations which on their own were not effective			X		
Simple checklists and indicators that reflect community experience and are observable by them			X	X	
Strategic orientation					
Working with communities and health workers to raise awareness of rights, rather than just one side			X		X
Fostering a common language, clarifying rules to counter power imbalances, fostering dialogue and mutual understanding, supporting a constructive rather than confrontational approach			X	X	X
Multi-level and multi-stakeholder initiatives that build synergies from household level interventions, community actions, health facility interventions to broader systems wide initiatives					X
Strategic planning and concrete operationalization					
Situational analysis of community, health care system, local governance and higher level policy and management linkages;			X	X	X
Time and capacity-building of all stakeholders; Iterative processes to support changed attitudes and norms				X	X

The poor availability and low quality of information in the 26 documents examined indicate that while numerous examples of promoting rights for quality maternity services exists, the capacity, time and resources to support research partnerships to systematically evaluate their processes, learning and effects is lacking.

### Strengths and limitations

Strengths of the synthesis include the concerted effort to triangulate multiple potential sources of information, to overcome the challenge of finding quality documentation of interventions that promote awareness of rights for maternity care services, despite consensus of its strategic value. Nonetheless, the variable quality across all documents meant an over-reliance on the few studies and reports that documented stakeholder perspectives and implementation considerations.

## Conclusion

Despite the diversity of examples found, initiatives promoting awareness of women’s rights to quality maternity care services have a strong commonality. They can have strong positive effects on individual women, nurturing collective dialogue and negotiation skills with other stakeholders to raise awareness of and respond to their rights. While illustrative examples are highlighted in this review, many interventions have yet to be evaluated and several are poorly documented. More investment is required to create and sustain research partnerships that support such initiatives and better elucidate their context specific variations. Rather than being aspirational, promoting awareness of women’s rights to quality maternity services is more than just disseminating information about rights. The strongest examples reflected efforts to change mindsets and relationships among varied stakeholders at different levels of the health system through social processes over time to overcome resistance, backlash and setbacks. Promoting and realizing these rights are at the heart of societal and health system transformation. They require sustained investment and are not for the feint hearted or weary.

## Abbreviations

ISOFI: Inner spaces outer faces initiatives
NGO: Non governmental organisation
SURE: Supporting the use of research evidence framework
WHO: World Health Organisation
